# Effects of individualized dietary counseling on nutritional status and quality of life in post-discharge patients after surgery for gastric cancer: A randomized clinical trial

**DOI:** 10.3389/fonc.2023.1058187

**Published:** 2023-02-27

**Authors:** Hongxia Yan, Fang He, Jianjian Wei, Qiuxiang Zhang, Chunguang Guo, Jinnv Ni, Fangyu Yang, Yingtai Chen

**Affiliations:** ^1^Department of Pancreatic and Gastric Surgery, National Cancer Center/National Clinical Research Center for Cancer/Cancer Hospital, Chinese Academy of Medical Sciences and Peking Union Medical College, Beijing, China; ^2^Department of Clinical Nutrition, Peking University Third Hospital, Beijing, China; ^3^School of Nursing, Capital Medical University, Beijing, China

**Keywords:** gastrectomy, dietary counseling, calorie intake, BMI loss, QOL

## Abstract

**Background:**

Currently, the supporting evidence for dietary counseling is insufficient. The aim of this study is to evaluate the impact of individualized dietary counseling on nutritional outcomes and quality of life (QOL) in patients undergoing surgery for gastric cancer.

**Methods:**

This study was a prospective, single-center, randomized controlled trial. The patients after surgery for gastric cancer were randomly assigned (1:1) to the intervention group and the control group. In the intervention group, patients receive individualized dietary counseling based on individual calorie needs and symptom assessment at 24 h before discharge, 14, 21, 30, and 60 days postoperatively. Patients in the control group received routine dietary counseling. The primary endpoint was body mass index (BMI) loss at 30, 60, and 90 days after surgery; the secondary endpoints were calorie and protein intake at 30 and 60 days after surgery, blood parameters, the 90-day readmission rate, and QOL at 90 days after surgery.

**Results:**

One hundred thirty patients were enrolled; 67 patients were assigned to the intervention group and 63 patients to the control group. Compared with the control group, patients in the intervention group were significantly less BMI loss at 30 days (−0.84 ± 0.65 vs. −1.29 ± 0.83), 60 days (−1.29 ± 0.92 vs. −1.77 ± 1.13), and 90 days (−1.37 ± 1.05 vs. −1.92 ± 1.66) after surgery (all *P<* 0.05). Subgroups analysis by surgery type showed that the intervention could significantly reduce BMI loss in patients undergoing total and proximal gastrectomy at 30 days (−0.75 ± 0.47 vs. −1.55 ± 1.10), 60 days (−1.59 ± 1.02 vs. −2.55 ± 1.16), and 90 days (−1.44 ± 1.19 vs. −3.26 ± 1.46) after surgery (all *P<* 0.05). At 60 days after surgery, calorie goals were reached in 35 patients (77.8%) in the intervention group and 14 patients (40.0%) in the control group (*P* = 0.001), and protein goals were reached in 40 patients (88.9%) in the intervention group and 17 patients (48.6%) in the control group (*P<* 0.001). Regarding the QOL at 90 days after surgery, the patients in the intervention group had a significantly lower level of fatigue, shortness of breath and stomach pain, better physical function, and cognitive function (*P<* 0.05).

**Conclusions:**

Post-discharge individualized dietary counseling is an effective intervention to reduce post-gastrectomy patient weight loss and to elevate calorie intake, protein intake, and QOL.

## 1 Introduction

Gastric cancer (GC) is the fifth most common type of cancer and the third most common cause of cancer-related death worldwide, seriously threatening human life and health (1). So far, treatment modalities include surgery, chemotherapy, and radiotherapy, and surgery remains the main and most effective therapy for GC (2). However, GC surgery has caused reduction of food storage volume and various gastrointestinal symptoms, threatening the nutritional status of patients to varying degrees (3–5). Body weight loss and malnutrition are frequently observed in patients who undergo gastrectomy (6). Malnutrition has been indicated to have negative influence patients’ clinical outcomes, including increased risk of recurrence, decreased tolerance to treatment, and quality of life (QOL) (7–10).

Home rehabilitation after GC surgery is a special period. Patients with reconstructed gastrointestinal tracts have just regained partial function and are still at suffering from post-surgical syndromes and the risk of readmissions (11). During this period, patients gradually transition from semi-liquid foods to soft or regular foods, and their diet is inevitably restricted (12). Because of the influence of gastrointestinal symptoms, some patients often take the reduced intake method to relieve gastrointestinal symptoms. Poor eating habits that lack high-quality protein in the diet. These factors can lead to insufficient calorie and protein intake (13). A Korean study confirmed that most patients after GC surgery experienced reduced food intake and rapid weight loss during this period (14). Therefore, appropriate nutritional support should be adopted to assist patients in a smooth transition to complete oral feeding, which has an important clinical significance for maintaining postoperative body weight and improving chemotherapy tolerance.

Previous studies have shown that oral nutritional supplement (ONS) and nutrition education are beneficial for patients with GC after surgery (15–18). However, there is a gap between the actual intake of ONS and the recommended amount due to factors such as intolerance (19, 20) and the role of dietary intervention and counseling is uncertain, and further research into optimal nutrition support interventions and timing of interventions is required (21, 22). In recent years, individualized dietary nutrition counseling strategies based on the individual calorie needs of patients have provided nutritional support to patients at nutritional risk, showing benefits on clinical outcomes of patients (23). The study was carried out only on patients who were hospitalized. Home-based dietary nutrition counseling is rarely reported due to the existence of many barriers, such as dietary restrictions, symptom burden, time and space inconvenience, and patient compliance.

In the present study, we aim to systematically evaluate the impact of individualized dietary counseling based on individual calorie and protein needs compared with routine discharge counseling follow-up on nutritional outcomes, the 90-day readmission rate, and QOL in post-discharge patients after GC surgery.

## 2 Methods

### 2.1 Study design and patients

This prospective, single-center, randomized controlled trial was conducted in the Department of Pancreatic and Gastric Surgery, National Cancer Center/National Clinical Research Center for Cancer/Cancer Hospital from August 2021 to January 2022. This study was approved by the hospital ethics committee, and the ethics approval number is 21/281-2952. After informed consent, patients were randomly allocated into individualized dietary counseling group (intervention group) and routine dietary counseling group (control group) on a 1:1 ratio through excel random number table. All patients’ calorie and protein calculations in the diet diary were calculated by masked nurses according to the China Food Composition Tables—Standard Edition (24).

We enrolled patients who were aged 18–75 years, discharged after radical gastrectomy, and reconstruction of gastrointestinal tract function recovery, allowing food intake and barrier-free communication. The patients were excluded if they were combined with other organ resections, had complications, and were not allowed to eat, such as anastomotic leakage, intestinal obstruction, and gastroparesis after surgery; and were in other nutritional intervention studies at the same time. Patients were rejected if they withdrew the informed consent, were lost to follow-up, were readmitted to the hospital and unable to eat for more than 72 h, were found with local recurrence of tumor, and received radiotherapy that seriously affected eating.

### 2.2 Study protocol

An individualized nutrition support team consisting of surgeons, nurses, and dieticians was set up. Surgeons were responsible for the treatment of gastrointestinal symptoms and diagnosis and treatment of readmissions; dieticians guided nurses to calculate calorie requirements, customize the recipe, and make dynamic adjustments; and nurses were the main executors of individualized dietary counseling.

All patients signed the consent form 24 h before discharge and collected baseline data. We collected patients’ general and clinical information including age, gender, education level, long-term residence, medical history, smoking status, alcohol consumption, type of surgery, surgery time (minutes), postoperative hospital stay (days), preoperative complications, American Joint Committee on Cancer (AJCC) stage, neoadjuvant chemotherapy, and adjuvant chemotherapy. A symptom assessment was conducted, and a dietary diary for 24 h was handed out. On discharge day, morning weight and dietary diary for 24 h are withdrawn. All patients received the first dietary counseling, the intervention group received individualized dietary counseling, and the control group received conventional dietary counseling.

**Figure 1:**
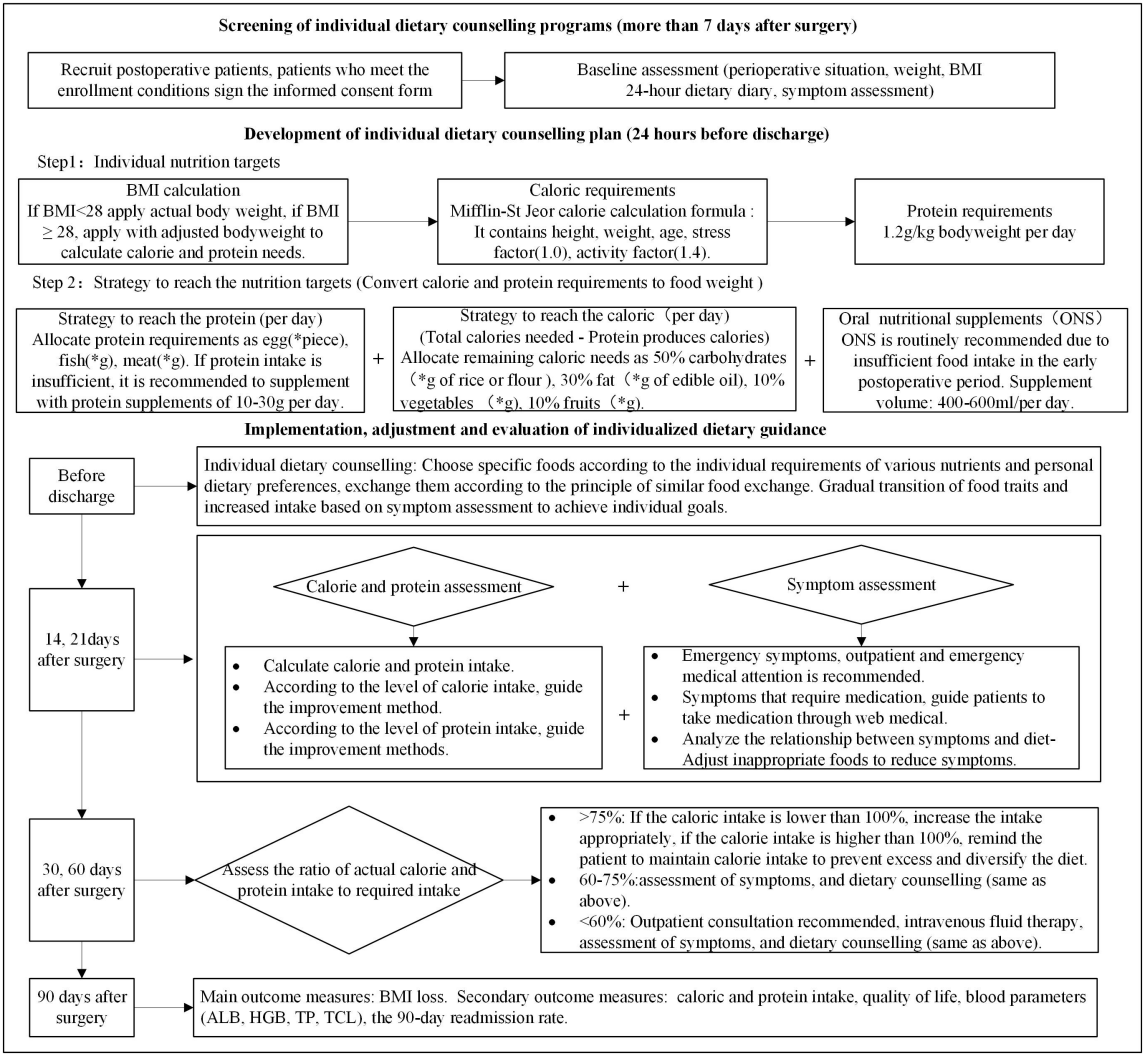
Intervention group study flow chart. Twenty-four–hour diet diary (self-designed form); symptom assessment (MDASI-C); “*”, calculate according to the patient’s needs.


In the intervention group, patients received individualized dietary counseling to reach protein and calorie goals, as shown in **Figure 1**. The daily protein requirement is set at 1.2 g/kg according to the nutritional guidelines (25). Calorie requirements were predicted using the Mifflin-St Jeor equation; it contains resting energy expenditure (REE), the stress factor, which is set to 1.0, and the activity factor, which is set to 1.4 (26); and the calculation method is as follows:

(1) $$TEE=REE\times factor_{activity}\times factor_{stress}$$

(2) $$\{\begin{array}{c} REE_{Men}=9.99\times Weight+6.25\times Height-4.92\times Age+5\\ REE_{Women}=9.99\times Weight+6.25\times Height-4.92\times Age-161 \end{array}$$

(3) $$Weight_{Adjusted}=Weight_{Ideal}+\left(Weight_{Actual}-Weight_{Ideal}\right)\times 0.4$$

In this study, if body mass index (BMI) ≤ 28, then actual body weight is applied; if BMI > 28, then adjusted body weight is applied to calculate calorie and protein needs. According to calorie and protein needs, it is converted into food in an appropriate proportion. When calories are converted into food, the protein requirements are first converted into foods such as fish, meat, eggs, beans, milk, and other foods and then the energy produced by protein foods is excluded; the remaining energy required is distributed: 50% of the calories comes from carbohydrates, 30% from lipids, 10% from vegetables, and 10% from fruits. ONS is routinely recommended because of the insufficient food intake in the early postoperative period, and the recommended dose is 400–600 ml per day (27). In this study, ONS is added in addition to food, so it does not occupy the total amount of calories needed. The intake is determined by the patient and is not mandatory. If protein intake is insufficient, then it is recommended to supplement with protein supplements of 10–30 g per day. It is recommended that patients cook semi-liquid or soft food before eating. The dietary plan was dynamically adjusted according to the actual calorie and protein intake in the 24-h diary, and symptom assessment was conducted at 14, 21, 30, and 60 days after surgery. The European Society for Parenteral and Enteral Nutrition (ESPEN) defines insufficient oral intake as an oral intake ≤75% of the estimated daily calorie needs (28). At 30 and 60 days after surgery, if the total oral intake (including food and ONS) is less than 60%, then intravenous supplementation is recommended. Patients with intakes in the range of 60%–75% received individualized adjustments based on patient diet and symptom assessment. Intakes greater than 75% were considered to achieve the target amount; if the calorie intake is lower than 100%, then the intake appropriately increases; if the calorie intake is higher than 100%, then the patient is reminded to maintain the current calorie intake to prevent excessive gastrointestinal burden and further diversify the diet.

Patients in the control group received conventional dietary counseling. It includes dietary principles, types, methods, and contraindications. ONS and protein powder can be supplemented, if necessary. If they encountered problems such as persistent vomiting and severe abdominal pain, then they were accessible to consultation with medical staff through WeChat, telephone, and outpatient or emergency medical treatment in time. Routine follow-up was performed 30 and 60 days after surgery, including 24-h dietary intake and symptoms assessment, and assisted with readmissions. There is no dynamic dietary modification regimen based on the patient’s calorie needs and actual calorie intake.

### 2.3 Endpoints

The primary endpoint was BMI loss at 30, 60, and 90 days postoperatively. Height measurement is uniformly measured by nurses at admission. Body weight (kilograms) was measured in the morning after defecation on an empty stomach; the patients are wearing light clothing and removed their shoes, cell phones, watches, etc. Post-discharge body weights were collected in the form of patient self-reports, and then, nurses calculated BMI.

The secondary endpoints were the 24-h calorie and protein intake at 30 and 60 days after surgery, blood parameters, the 90-day readmission rate, and QOL at 90 days after surgery. This study used a prospective dietary record approach, and food was weighed for each meal at 30 and 60 days after surgery. Blood parameters including total protein (TP), albumin (ALB), hemoglobin (HGB), and total lymphocyte count (TLC). Ninety-day readmission rates (unplanned readmission within 90 days of surgery) and QOL (EORTC QLQ-C30 and EORTC QLQ-STO22) were assessed at 90 days after surgery. Loss of follow-up, completion of diet records at each follow-up, and completion of review at 90 days after surgery were used as indicators to evaluate compliance.

### 2.4 Data collection

We assessed QOL with the European Organization for Research and Treatment of Cancer (EORTC) generic cancer (QLQ-C30) and GC (QLQ-STO22) modules (29). Patients complete QOL assessment *via* electronic questionnaire at 90 days after surgery. EORTC QLQ-C30 is a reliable and validated measure of the QOL of patients with cancer in multicultural clinical research. The questionnaire is a cancer-specific, self-administered, structured questionnaire that contains 30 questions, which are categorized into the global health status; five functioning scales: physical, role, cognitive, emotional, and social; three symptom scales: fatigue, pain, and nausea and vomiting; and six single items: dyspnea, sleep disturbance, appetite loss, constipation, diarrhea, and financial difficulties. The GC module (QLQ-STO22) is a supplement to the QLQ-C30. The QLQ-STO22 consists of 22 questions that evaluate five multi-item symptoms scales (dysphagia, eating restrictions, pain, reflux, and anxiety) and four single-item symptoms scales (dry mouth, body image, hair loss, and taste loss). For global QOL and the functional scales, a higher score indicates better QOL, with 100 being perfect. For symptom scales, a lower score indicates better QOL, with 0 being perfect or no symptoms reported.

Symptoms were assessed using the Chinese version of the M. D. Anderson Symptom Inventory–China (MDASI-C). Scale items are calculated on a scale of 1–10, with 0 indicating no influence, and 10 indicating severe influence. MDASI-C has good internal consistency reliability, general symptoms, and gastrointestinal symptoms, which had Cronbach alpha coefficients of 0.86 and 0.84 (30). This study only used this scale to evaluate gastrointestinal symptoms and provide dietary counseling to patients, and symptom score is not used as an outcome measure.

### 2.5 Sample size and statistical analysis

In this study, two independent sample mean comparison superiority experiments were used to calculate the sample size. On the basis of the previous RCT study (18), the postoperative weight loss (−6.9 kg vs. −9.1 kg) was calculated, taking into account the loss rate and dropout rate of 20%; the sample size was estimated to be about 130 cases; and the intervention group and the control group took a 1:1 ratio entry.

SPSS 25.0 statistical software was used for data analysis. Mean ± standard deviation, median, and quartile were used to describe the characteristics of continuous data, and frequency, rate, and percentage were used for count data. Differences between the two groups were presented through a t-test or nonparametric test, and a chi-square test was used for univariate analysis. Repeated-measures ANOVA were used to test the between-group, within-group, and interaction effects of repeated-measures measurement data. A two-sided test was used, and *P<* 0.05 was considered statistically significant.

## 3 Results

### 3.1 Patient characteristics

**Figure 2:**
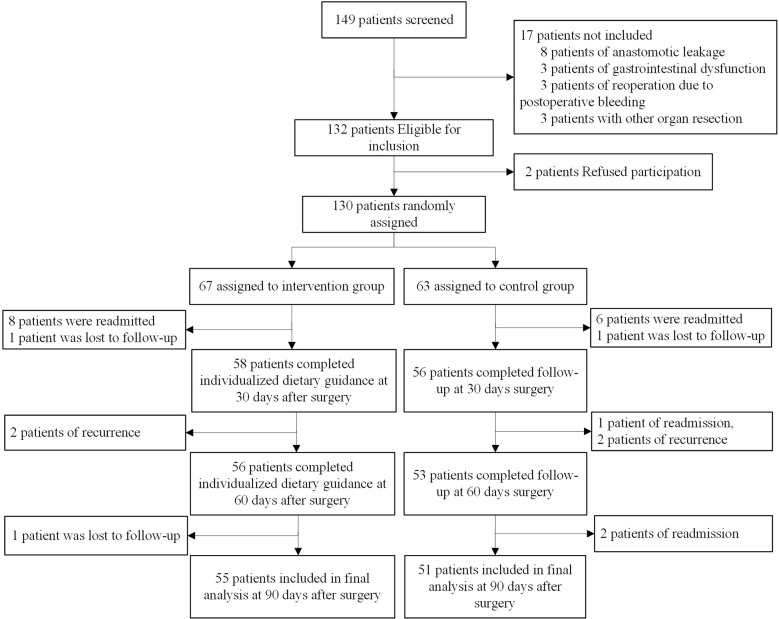
Trial profile.

From August 2021 to January 2022, we screened 149 patients and enrolled 130, 17 patients were excluded according to the exclusion criteria, and two patients refused to participate. Of these, 67 patients were randomly assigned to the intervention group and 63 to the control group. In our study, 21 patients were shaved, 17 patients fasted for more than 72 h due to readmission, and four underwent postoperative radiotherapy due to local recurrence, which severely affected eating. Three patients (2.31%) were lost to follow-up. Our final evaluable cohort consisted of 106 patients (55 patients in the intervention group and 51 patients in the control group), as shown in **Figure 2**.

There was no significant difference in baseline data between the two groups except for preoperative comorbidities (*P* > 0.05), as shown in **Table 1**. The compliance of dietary records in the intervention group was higher than that in the control group at 30 days after surgery (*P* = 0.048), as shown in **Table 2**.

**Table 1:** General characteristics of gastrectomy patients at baseline.

Characteristics	Variable	Intervention (n = 67)	Control (n = 63)	*P-*value
Age (years)	/	56.16 ± 11.42	57.40 ± 12.07	0.551
Gender	Male/Female	42/25	36/27	0.519
Long-term residence	City	42	36	0.519
	Town and country	25	27	
Education	Elementary school	6	11	0.058
	Middle school	32	37	
	Technical secondary school	10	8	
	University and above	19	7	
Preoperative BMI	/	22.86 ± 2.77	23.40 ± 3.38	0.323
Preoperative PG-SGA	/	4.03 ± 3.31	5.05 ± 3.51	0.093
Smoking	Yes/No	47/20	35/28	0.082
Alcohol intake	Yes/No	50/17	45/18	0.681
Preoperative comorbidities	Yes/No	14/53	29/34	0.002
Hypertension	Yes/No	8/59	18/45	0.018
Diabetes	Yes/No	6/61	10/53	0.230
Heart disease	Yes/No	2/65	4/59	0.361
Surgical approach	Open	4	4	0.739
	Laparoscopic assisted	50	50	
	Total laparoscopic	13	9	
Type of surgery	DG	45	47	0.351
	TG and PG	22	16	
Surgery time (minutes)	/	202.27 ± 48.73	195.10 ± 63.93	0.472
Postoperative hospital stay (days)	/	11.21 ± 3.81	10.62 ± 4.02	0.392
Postoperative complications	Yes/No	7/60	7/56	0.903
AJCC stage	0, I	25	30	0.185
	II	8	11	
	III	34	22	
Neoadjuvant chemotherapy	Yes/No	14/53	13/50	0.971
Postoperative chemotherapy	Yes/No	40/27	35/28	0.633

Values are presented as mean ± standard deviation or number. t-test and chi-square test.

Postoperative complications: abdominal infection and lung infection.

**Table 2:** Evaluation of patient compliance.

Variable	Intervention (yes/no)	Control (yes/no)	*P*
Lost to follow-up	2/65	1/62	0.587
Unrecorded diet (30 days after surgery)	3/52	9/42	0.048
Unrecorded diet (60 days after surgery)	10/45	16/35	0.115
Not reviewed (90 days after surgery)	12/42	9/42	0.757

### 3.2 Nutritional outcomes

There were no significant differences in the mean BMI between the two groups before discharge, 30, 60, and 90 days after surgery (*P* > 0.05). Repeated-measures ANOVA (BMI at discharge was used as the covariate) for BMI loss in the two groups showed that there was a statistically significant difference between the within-subject effect (*P* = 0.001), the between-subject effect (*P* = 0.026), and the interaction effect (*P* = 0.051). Compared with the control group, patients in the intervention group have significantly less BMI loss at 30 days (−0.84 ± 0.65 vs. −1.29 ± 0.83, *P* = 0.002), 60 days (−1.29 ± 0.92 vs. −1.77 ± 1.13, *P* = 0.020), and 90 days (−1.37 ± 1.05 vs. −1.92 ± 1.66, *P* = 0.044) after surgery, as shown in **Table 3**.

**Table 3:** Evaluation of patient weight and BMI.

Time	Variable	Intervention	Control	*P*
Discharged	Weight	64.44 ± 11.12	64.31 ± 12.23	0.957
	BMI	22.79 ± 2.69	23.59 ± 3.66	0.201
30 days after surgery	Weight	62.04 ± 10.52	60.85 ± 11.47	0.578
	Weight loss	−2.39 ± 1.88	−3.46 ± 2.27	0.009
	BMI	21.95 ± 2.58	22.30 ± 3.33	0.551
	BMI loss	−0.84 ± 0.65	−1.29 ± 0.83	0.002
60 days after surgery	Weight	60.75 ± 10.34	59.60 ± 11.45	0.589
	Weight loss	−3.69 ± 2.72	−4.71 ± 2.99	0.067
	BMI	21.49 ± 2.54	21.82 ± 3.25	0.561
	BMI loss	−1.29 ± 0.92	−1.77 ± 1.13	0.020
90 days after surgery	Weight	60.53 ± 10.70	59.26 ± 11.52	0.558
	Weight loss	−3.91 ± 3.05	−5.05 ± 4.36	0.118
	BMI	21.41 ± 2.69	21.67 ± 3.12	0.653
	BMI loss	−1.37 ± 1.05	−1.92 ± 1.66	0.044

**Figure 3:**
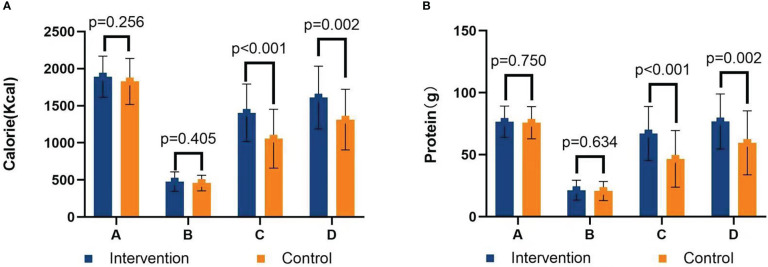
**(A)** Graph of intervention on BMI loss after total gastrectomy and proximal gastrectomy. **(B)** Graph of intervention on BMI loss after distal gastrectomy. A=Discharge day; B=30 days after surgery; C=60 days after surgery; D=90 days after surgery.

Subgroups analysis by surgery type showed that the intervention could significantly reduce BMI loss in patients undergoing total gastrectomy (TG) and proximal gastrectomy (PG) at 30 days (−0.75 ± 0.47 vs. −1.55 ± 1.10, *P* = 0.013), 60 days (−1.59 ± 1.02 vs. −2.55 ± 1.16, *P* = 0.025), and 90 days (−1.44 ± 1.19 vs. −3.26 ± 1.46, *P* = 0.001) after surgery, as shown in **Figure 3A**. However, the intervention for patients undergoing distal gastrectomy (DG) was only different at 30 days after surgery (−0.88 ± 0.72 vs. −1.21 ± 0.71, *P* = 0.046), and there was no significant difference at 60 days (−1.17 ± 0.86 vs. −1.49 ± 1.00, *P* = 0.133) and 90 days (−1.35 ± 0.99 vs. −1.46 ± 1.47, *P* = 0.691) after surgery, as shown in **Figure 3B**.

**Figure 4:**
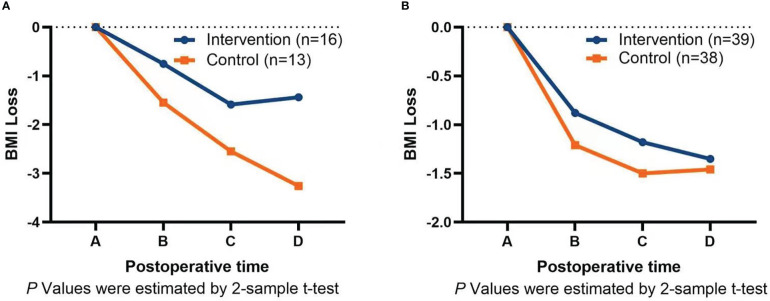
**(A)** Calorie intake chart. **(B)** Protein intake chart. A=Calorie/protein requirements; B=24-h calorie/protein intake before discharge; C=30 days calorie/protein intake after surgery; D=60 days calorie/protein intake after surgery.

There was no significant difference in calorie and protein requirements and intake before discharge between the two groups (*P* s> 0.05). Compared with patients in the control group, patients in the intervention group had significantly higher calorie intake (1,404.75 ± 387.40 kcal vs. 1,056.33 ± 396.22 kcal at 30 days after surgery, *P<* 0.001; 1,611.11 ± 423.11 kcal vs. 1,313.11 ± 408.26 kcal at 60 days after surgery, *P* = 0.002), as shown in **Figure 4A**. The protein intake in the intervention group was significantly higher than that in the control group (67.15 ± 21.78 g vs. 46.71 ± 22.89 g at 30 days after surgery, *P<* 0.001; 76.89 ± 22.21 g vs. 59.59 ± 25.84 g at 60 days after surgery, *P* = 0.002), as shown in **Figure 4B**. Although ONS was recommended, patients’ intake was not satisfactory. The ONS intake in the intervention group was higher than that in the control group (163.65 ± 154.16 ml vs. 79.77 ± 112.32 ml at 30 days after surgery, *P* = 0.003; 123.11 ± 148.41 ml vs. 43.25 ± 74.85 ml at 60 days after surgery, *P* = 0.003).

At 30 days after surgery, calorie goals were reached in 25 patients (48.1%) in the intervention group and five patients (11.9%) in the control group (*P<* 0.001), and protein goals were reached in 36 patients (69.2%) in the intervention group and 12 patients (40.0%) in the control group (*P<* 0.001). Calorie intake was less than 60% in 11 patients (21.2%) in the intervention group and 23 patients (54.8%) in the control group (*P* = 0.001), and the patients were advised to supplement energy through an intravenous route on the basis of oral diet, but only two patients received intravenous supplementation. At 60 days after surgery, calorie goals were reached in 35 patients (77.8%) in the intervention group and 14 patients (40.0%) in the control group (*P* = 0.001), and protein goals were reached in 40 patients (88.9%) in the intervention group and 17 patients (48.6%) in the control group (*P<* 0.001). Calorie intake was less than 60% in 4 patients (8.9%) in the intervention group and nine patients (25.7%) in the control group (*P* = 0.043); however, no patient received intravenous supplementation therapy, and three patients used traditional Chinese medicine.

Blood parameters showed that there was no significant difference in TP, serum ALB, HGB, and TLC between the two groups before discharge and 90 days after surgery (*P* > 0.05), as shown in **Table 4**.

**Table 4:** Evaluation of patient blood parameters.

Time	Index	Intervention (n = 55)	Control (n = 51)	*P*
Before discharge	TP	60.99 ± 4.94	58.31 ± 6.81	0.078
	ALB	34.64 ± 3.03	34.08 ± 3.55	0.387
	HGB	112.73 ± 18.14	112.96 ± 15.47	0.994
	TLC	1.38 ± 0.58	1.41 ± 0.58	0.775
90 days after surgery	TP	70.71 ± 3.36	66.99 ± 4.43	0.653
	ALB	42.93 ± 1.88	40.39 ± 3.26	0.494
	HGB	124.95 ± 16.47	122.67 ± 18.74	0.657
	TLC	1.96 ± 0.48	1.92 ± 0.68	0.367

Data are expressed as means ± standard deviation.

TP, total protein (g/L); ALB, albumin (g/L); HGB, hemoglobin (g/L); TLC, total lymphocyte count (*10^9^/L).

### 3.3 Ninety-day readmission rate

During the course of the study, eight patients in the intervention group had readmission, and nine patients in the control group had readmission within 90 days after surgery; there was no statistically significant difference (*P* > 0.05). The specific reasons for readmission are shown in **Table 5**.

**Table 5:** Reasons for readmission within 90 days after surgery.

Reason for readmission	Intervention (n = 67)	Control (n = 63)	Total
Gastrointestinal dysfunction	3	4	7
Abdominal infection	2	0	2
Bleeding	1	1	2
Intestinal obstruction	0	2	2
Heartburn reflux	1	1	2
Diarrhea	1	0	1
Cholecystitis	0	1	1
Total	8	9	17

For the outcomes of readmission patients, among them, 14 patients improved after infusion therapy, one patient with intestinal obstruction improved after surgery, one bleeding patient improved after emergency surgery for hemostasis, and one bleeding patient died after interventional hemostasis.

### 3.4 Quality of life

Regarding the QOL at 90 days after surgery, the patients in the intervention group had a significantly lower level of fatigue, shortness of breath, and stomach pain; and better physical function and cognitive function (*P<* 0.05), as shown in **Table 6**.

**Table 6:** Evaluation of patient QOL at 90 days after surgery.

	Intervention (n = 55)	Control (n = 51)	*P*
EORTC-QLQ-C30			
Global health	75 (58, 83)	66 (50, 83)	0.156
Physical function	93 (86, 100)	86 (73, 86)	0.001
Role function	100 (66, 100)	83 (66, 100)	0.174
Emotional function	66 (58, 75)	66 (50, 75)	0.786
Cognitive function	100 (83, 100)	83 (66, 100)	0.006
Social function	83 (66, 100)	66 (66, 100)	0.115
Fatigue	0 (0, 22)	22 (0, 33)	0.003
Nausea and vomiting	0 (0, 16)	0 (0, 33)	0.116
Pain	0 (0, 0)	0 (0, 0)	0.393
Dyspnea	0 (0, 0)	0 (0, 33)	0.002
Insomnia	0 (0, 33)	0 (0, 33)	0.069
Appetite loss	0 (0, 33)	0 (0, 33)	0.388
Constipation	0 (0, 0)	0 (0, 0)	0.611
Diarrhea	0 (0, 0)	0 (0, 0)	0.087
Financial difficulties	0 (0, 33)	33 (0, 66)	0.054
EORTC-QLQ-STO22			
Dysphagia	0 (0, 0)	0 (0, 0)	0.783
Abdominal pain	0 (0, 8)	8 (0, 25)	0.014
Reflux symptoms	0 (0, 11)	0 (0, 11)	0.207
Eating restrictions	0 (0, 0)	0 (0, 0)	0.592
Anxiety	22 (0, 33)	22 (0, 33)	0.471
Having dry mouth	0 (0, 0)	0 (0, 33)	0.423
Taste	0 (0, 0)	0 (0, 33)	0.105
Body image	0 (0, 0)	0 (0, 0)	0.806

Data are shown as the median (p25, p75).

## 4 Discussion

It is well known that malnutrition after GC surgery is closely associated with poor prognosis and decreased QOL. For a long time, dietary counseling has been performed to improve the nutritional status of patients after a gastrectomy; it is regarded as an essential and valuable tool for influencing nutritional status. However, the effect of dietary counseling on the nutritional status of patients after GC surgery is not clear. The patients in our study received individualized dietary counseling, and each patient’s nutritional goals and required nutritional support were individually defined. Therefore, our study provides evidence that an overall strategy of providing individualized dietary counseling based on calorie and protein requirements and a symptom assessment to achieve protein and calorie goals during postoperative recovery are beneficial to patients. Our findings validate some previous trials (15, 16) but contradict the findings of the meta-analysis (22), which reported that all nutritional counseling studies did not show significant differences. Another meta-analysis also reported, finding very low-quality evidence to support the effect of oral nutritional interventions on post-hospital weight and energy or protein intake (21). However, there was no significant difference in 90-day readmission rates and blood parameters, which is also consistent with previous nutritional intervention studies (17).

Many studies have shown that, after TG, patients with more weight loss and more nutritional problems are more significant (31, 32). Therefore, the effect of intervention measures on patients with TG will be more concerned. Subgroups analysis by surgery type showed that the intervention could significantly reduce BMI loss in patients undergoing TG and PG at 30, 60, and 90 days after surgery. This is similar to the results of the related nutritional intervention study in Japan (18, 33), which showed that oral enteral nutrition intervention in patients after GC surgery had a significant effect in patients with TG, but there was no difference in patients undergoing DG. Analysis of the reasons may be that patients with TG and PG face greater nutritional risks and require more scientific diet and nutritional interventions to meet their physical needs. Compared with TG and PG, patients undergoing DG face lower nutritional risks (34), and most patients can gradually adapt to postoperative changes in the gastrointestinal tract by adjusting their diets. Only in the early postoperative stage that some patients with severe gastrointestinal symptoms require further individualized counseling and management.

With respect to the QOL, it is always a major concern for the prognostic after nutritional support. Related research in Korea shows that follow-up management on nutritional intervention for patients undergoing gastrectomy will have a positive impact on their QOL (15). Our research also supports these results, and nutritional status is closely related to QOL. Relevant studies in the United States have identified HRQOL issues related to dietary changes and restrictions after upper gastrointestinal cancer treatment, involve family caregivers, and are tailored and flexible to patient and family caregiver’s needs and preferences (35). The recommendations of this study’s findings were fully considered in our study design. While the underlying reason for the effect on the QOL was not investigated in the present study, we speculated that the reduced fatigue, shortness of breath, and stomach pain may be associated with individualized dietary counseling to reasonably adjust food types and intake based on symptom assessment, and increased calorie and protein intake was associated with improved physical function and cognitive function.

Several points of this trial are worth mentioning. First, in our study, the patient was recovering from major abdominal surgery, and the calorie intake of the patients was about 25% of the required amount at the time of discharge. All patients had NRS2002 scores of 4–5 with nutritional risk, so it was necessary to develop an individualized nutritional treatment plan based on clinical practice. Our research philosophy is to address the recent postoperative food intake restriction by improving food quality to improve the nutritional status of patients. This study is a comprehensive intervention plan that includes concepts of dietary counseling, ONS, symptom management, and ongoing management. Second, dynamic adjustment is made according to the patient’s dietary habits and actual intake of nutrients. The results of a Korean study show that simplified dietary education is ineffective for patients after GC surgery. It is very challenging to influence patients’ dietary habits to improve their nutritional status in surgical patients, especially after gastrectomy, and points out the need for consultation sufficient time, appropriate materials to support, and iterative and regular feedback (36). Therefore, we adjust the composition of various nutrients in the food according to the patient’s actual food intake type and intake and increase the content of calories and protein in food as much as possible under the same tolerated volume. This adjustment was repeated, with at least five feedback and adjustments per patient. We help patients to form a high-protein, high-calorie, and small-volume dietary pattern suitable for the characteristics of the postoperative gastrointestinal tract. Third, regarding the time frequency of intervention and adjustment, according to the results of previous studies, the weight loss of patients with GC was the largest within 90 days after surgery, especially within 30 days after surgery (6, 14, 37). Therefore, the duration of intervention in this study was determined to be within 90 days after surgery, which is a critical period for the reconstruction of patients’ dietary patterns. The main adjustment and feedback frequency are determined to be once a week within 30 days after surgery, once a month after the 30th day, and until the 90th day after surgery; particular questions can be consulted through the online clinic at any time. Finally, no specific adverse side effects of the intervention were observed in our study. To ensure the safety of patients, patients’ compliance was evaluated by the degree of completion of home dietary guidance and postoperative review (mainly includes loss of follow-up, whether to keep a food diary, and whether to conduct postoperative review at 90 days after surgery), and dietary intake was not used for compliance evaluation. We only set calorie and protein targets for patients and instruct and assist patients in adjust the amount of each meal, food characteristics, and cooking methods according to the symptoms after eating. We also applied the MDASI-C to truly reflect the burden of digestive tract symptoms of patients, screened the symptoms of emergency readmission and recommended prompt medical treatment, and stratified management of other minor symptoms, ensuring patients’ safety.

## 5 Prospect of further research

First prospect of future research is to explore the mechanism of postoperative weight loss in patients with GC. In recent years, epigenetic studies have shown that many dietary components may indirectly influence genomic pathways for DNA methylation, and there is evidence of a biochemical link between nutritional quality and mental health (38). Precision nutrition is an emerging area of nutrition research, with a primary focus on the individual variability in response to dietary and lifestyle factors (39). Second, intelligent terminal products are developed, and the convenience of artificial intelligence and network is utilized to match the food types, the intake of each food, and the gastrointestinal symptoms of patients, so as to provide quantitative and dynamic whole-life nutrition management for postoperative patients with GC and to improve the overall nutritional status and QOL of patients with GC. Third, symptom management was used as the main method of nutritional intervention in patients with GC after operation to explore its influence on nutritional status and QOL.

We are aware of the limitations of our study. First, although the primary results at 30, 60, and 90 days were objective, and calorie and protein intake calculations were masked, and some of the outcomes assessed during the home setting might have been vulnerable to observer (patient and caregiver) bias. Second, three (5.5%) patients in the intervention group and nine (17.6%) patients in the control group did not record their diet as required, despite implementation of the post-discharge individualized dietary counseling by trained nurses 30 days after surgery, and the number of patients further increased at 60 days after surgery. Similar to real-life experience, several patient, treatment, and hospital factors (for example, patient delay or refusal to initiate enteral or parenteral nutrition, and interference of chemotherapy with nutritional support) might have prevented full adherence to the protocol. Third, chemotherapy starts at 30 days after surgery, and the impact of chemotherapy on patients cannot be tracked in detail. Although data collection should be avoided during the chemotherapy period and within 7 days after chemotherapy, the delayed side effects of chemotherapy drugs may affect patients. Fourth, the sample size of our study was small, and the observation period was short. Although individualized dietary counseling is too complex for an expanded population, some variables may be overlooked due to the small number of cases. Fifth, some patients were unable to return to the National Cancer Center for review at 90 days after surgery. Therefore, the study lacked body composition (fat content and muscle content), body measurements (waist circumference, arm circumference, etc.), and PG-SGA scores that required face-to-face assessment. Only changes in BMI and food intake reflect nutritional status. Finally, in our study, because of the short intervention time and limited research funds, epigenetics and postoperative metabolic changes (blood glucose, blood pressure, and blood lipid) and other related results were not collected. We did not yet investigate the costs of the intervention, but we have planned to do a future cost-effectiveness analysis on the basis of the trial data (researcher’s time cost, economic cost, etc.); it is planned to adopt artificial intelligence assistance system to benefit more patients.

In conclusion, the trial showed that early use of individualized dietary guidance to help patients achieve protein and calorie goals after GC surgery could effectively increase energy and protein intake, reduce BMI loss, and improve QOL. The nutritional problems of patients after GC surgery are severe and complex. Further multi-center individualized dietary counseling research is needed to determine individualized interventions for different population characteristics, different surgical procedures, and different symptom burdens so as to improve the nutritional status of patients after GC surgery.

## Data availability statement

The raw data supporting the conclusions of this article will be made available by the authors, without undue reservation.

## Ethics statement

This study was approved by the hospital ethics committee, and the ethics approval number is 21/281-2952. The patients/participants provided their written informed consent to participate in this study.

## Author contributions

All authors made substantial contributions to the intellectual content of this paper. HY: protocol design, patient management, data analysis, and manuscript writing; FH: program design and supervision; JW: data collection and manuscript writing; QZ: program design and nutritional guidance; CG: medical processing and data analysis; JN: patient management, data collection, and collation; FY: supervision and critical review; YC: protocol design, supervision, and critical review. All authors contributed to the article and approved the submitted version.
